# Using deep learning to predict the hand-foot-and-mouth disease of enterovirus A71 subtype in Beijing from 2011 to 2018

**DOI:** 10.1038/s41598-020-68840-3

**Published:** 2020-07-22

**Authors:** Yuejiao Wang, Zhidong Cao, Daniel Zeng, Xiaoli Wang, Quanyi Wang

**Affiliations:** 1 The State Key Laboratory for Management and Control of Complex Systems, Institute of Automation, Chinese Academy of Sciences, Beijing, 100190 China; 2 University of Chinese Academy of Sciences, Beijing, 100049 China; 3 Institute for Infectious Disease and Endemic Disease Control, Beijing Center for Disease Prevention and Control, Beijing, 100013 China

**Keywords:** Infectious diseases, Computer science

## Abstract

Hand-foot-and-month disease (HFMD), especially the enterovirus A71 (EV-A71) subtype, is a major health problem in Beijing, China. Previous studies mainly used regressive models to forecast the prevalence of HFMD, ignoring its intrinsic age groups. This study aims to predict HFMD of EV-A71 subtype in three age groups (0–3, 3–6 and > 6 years old) from 2011 to 2018 using residual-convolutional-recurrent neural network (CNNRNN-Res), convolutional-recurrent neural network (CNNRNN) and recurrent neural network (RNN). They were compared with auto-regressio, global auto-regression and vector auto-regression on both short-term and long-term prediction. Results showed that CNNRNN-Res and RNN had higher accuracies on point forecast tasks, as well as robust performances in long-term prediction. Three deep learning models also had better skills in peak intensity forecast, and CNNRNN-Res achieved the best results in the peak month forecast. We also found that three age groups had consistent outbreak trends and similar patterns of prediction errors. These results highlight the superior performance of deep learning models in HFMD prediction and can assist the decision-makers to refine the HFMD control measures according to age groups.

## Introduction

HFMD is a mild gastrointestinal disease, mainly caused by EV-A, EV-B and EV-C species, while the EV-A71 subtype is prone to more serious complications^1^. Spatial and temporal patterns of HFMD incidence are strongly correlated with climatic factors^2–4^ , e.g. high-level humidity and middle-level temperatures. Social factors also affect the spread of HFMD, e.g. contact amongst children in school^5^. Under the influences of multi-type pathogenic viruses, complex climatic and social factors, HFMD presents a periodic outbreak in the Asia–Pacific region^1,5^. In China, the incidence and mortality of HFMD have been leading the type C infectious diseases since its severe outbreak in 2008 and the situation is getting worse. Beijing city, the capital of China, is also vastly threatened by HFMD^6^. Studies have explored the predominant virus^6,7^, weather factors^8,9^ and space–time patterns^10^ of HFMD in Beijing. Vaccines that prevent EV71-associated HFMD have been developed, but HFMD wouldn’t be eliminated because of many other pathogens. Therefore, forecasting the prevalence of HFMD in Beijing is still essential for public health.

Many previous studies used regressive models to predict the incidence of HFMD^11–16^. In addition to the HFMD incidence data, search index, temperature records, air quality and other exogenous variables were applied to fit the regressive models. Although these methods had achieved acceptable prediction accuracies, there are still some limitations. First, these works only focused on the total number of cases. They ignored the intrinsic age groups in children. In fact, children aged 0 to 6 are the most susceptible to HFMD, while children over the age of 6 have stronger immunities to HFMD. So, under the effect of epidemic transmission dynamics and immunity, there are connections among incidences in different age groups, i.e. 0–3, 3–6, > 6 years old, and they should be predicted simultaneously to leverage their relationships. The second limitation is that regressive models in previous studies are essentially linear models and their model capacities are insufficient to fit the complex multivariable dependencies. Since the peak magnitudes and peak months of HFMD are varying every year, we need nonlinear models to learn and predict their long-term dependencies.

Literature has shown a trend of using deep learning models to predict infectious diseases and overcome shortcomings in regressive models. Wang et al. applied a long-short term memory (LSTM) network to demonstrate its feasibility in HFMD prediction^17^. Further, some researches combined CNN, RNN and residual neural network into hybrid models^18^, to forecast influenza-like illness in Japan or USA^19–22^. With multiple exogenous variables as input, such as the number of cases or Google search indexes in different regions, deep learning models can simultaneously output the predictions of multiple regions with higher accuracy. The nonlinear function relationship in high-dimensional spatiotemporal data can be well learned. But the prediction of HFMD hasn’t been benefited from these hybrid models.

This study aims to forecast the prevalence of HFMD cases of EV-A71 subtype in Beijing from 2011 to 2018 using three deep learning models: RNN, CNNRNN and CNNRNN-Res. In particular, we (1) forecast the cases in three age groups (age 0–3, age 3–6, age > 6) and the total number of EV-A71 cases simultaneously, (2) compared the three deep learning models with three regressive models, i.e. AR, VAR and GAR, and (3) evaluated these six models in both short-term and long-term predictions. This study verified the advantages of deep learning models in the forecast of HFMD and indicated the association of incidences among three age groups, which can be used as additional information on HFMD prediction.

## Results

### Data Sources and Characteristics

**Figure 1:**
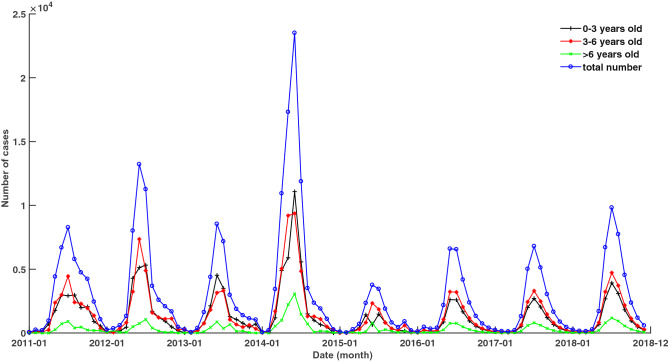
The monthly number of EV-A71 cases of three age groups in Beijing from 2011 to 2018. Black cross dots represent age group of 0–3; red dots represent age group of 3–6; green cross dots represent age group of > 6; blue circles represent the total number of EV-A71 cases.

The original data was the hospital visit data of EV-A71 virus infection, from the Beijing hand-foot-mouth disease surveillance system, and the time range was 2011–2018. To reasonably estimate the number of EV-A71 cases in the Beijing population, hospital data and population serum sampling data were combined for statistical inference using stratified inference^23^. Besides, Beijing has gradually inoculated the EV-A71 vaccines in the population since August 2016. As of December 2018, the total number of vaccinations was 298,341, which led to a significant decline in the number of EV-A71 cases in Beijing after 2016. Therefore, the prevalence of EV-A71 under non-vaccination after 2016 was reconstructed using a disease transmission kinetic model^24^. Figure 1 represents the monthly numbers of EV-A71 cases of three age groups in Beijing population from 2011 to 2018, after the statistical inferences and reconstruction. The number of EV-A71 cases showed a single peak or two peaks in a year and the largest outbreak was in 2014. The rapid increase of cases started in April and the peak month was June or July. After August, there were still small outbreaks and the decline was slow. Cases in the age group of > 6 were significantly less than the cases in the age groups of 0–3 and 3–6. As for the peak months, from 2011 to 2013, there were one-month lags in peak months between the age groups of 0–3 and 3–6. In 2015, both May and July were peak months in the age group of 0–3, while the peak month in the age group of 3–6 was June.

This dataset was split into a training set, validation set and test set in chronological order according to the ratio of 3:1:1. Each data subset was four-dimensional, including the number of cases in three age groups and the total number of cases. The training set (from January 2011 to September 2015) was responsible for model optimization. The test set (from May 2017 to December 2018) was used to measure the prediction accuracies of six models. And the role of validation set (October 2015 to April 2017) will be explained in detail in the Method section.

### Accuracy assessment on point forecasts

Tables 1 and 2 show the R-squares of three deep learning models (CNNRNN-Res, CNNRNN and RNN) and three regressive models (AR, VAR and GAR), respectively, with prediction horizons = 1, 2, 4, 6, 8, 10, 12 months. When horizon = 1 or 2, R-squares reflect the accuracies in short-term prediction; when horizon = 4, 6, 8, 10 and 12, R-squares can reflect the performance changes of the six models in long-term prediction.

**Table 1:** R-squares of CNNRNN-Res, CNNRNN and RNN predictions on test set in different age groups and horizons.

Model	Age group	Horizon (months)						
		1	2	4	6	8	10	12
CNNRNN-Res	0–3	0.8041	**0.7239**	0.3660	0.6908	0.4632	**0.8492**	**0.9032**
	3–6	**0.9174**	**0.8908**	0.4038	**0.7711**	**0.6889**	**0.8512**	0.8218
	> 6	**0.8823**	**0.8651**	0.3447	**0.7185**	**0.7779**	0.7730	0.7057
	Total	**0.9062**	**0.8467**	0.4647	**0.7654**	0.6274	0.8417	0.8538
CNNRNN	0–3	**0.8819**	0.5745	0.3596	0.3093	0.3734	0.8075	0.8715
	3–6	0.8769	0.6853	0.5535	0.4365	0.3966	0.7682	**0.8310**
	> 6	0.8306	0.6543	0.5927	0.3844	0.4263	0.6798	**0.7523**
	Total	0.8845	0.6659	0.5110	0.4244	0.4682	0.8106	**0.8661**
RNN	0–3	0.8662	0.4861	**0.4919**	**0.6955**	**0.7738**	0.8208	0.8291
	3–6	0.8881	0.6684	**0.6101**	0.7049	0.6855	0.8250	0.7032
	> 6	0.8615	0.6754	**0.6190**	0.6969	0.5862	**0.7849**	0.6464
	Total	0.8911	0.6302	**0.5913**	0.7255	**0.7292**	**0.8442**	0.7722

The number in bold indicates the maximum value in a certain horizon and age group. ‘Total’ means the total number of cases of EV-A71 subtype in Beijing.

**Table 2:** R-squares of AR, VAR and GAR predictions on test set in different age groups and horizons.

Model	Age group	Horizon (months)						
		1	2	4	6	8	10	12
AR	0–3	0.7538	0.3072	0.2518	**0.5693**	0.2776	0.3276	**0.4583**
	3–6	0.8505	**0.5976**	-0.4590	0.3501	-0.409	**0.4777**	0.4380
	> 6	0.7510	0.4755	0.3779	0.2717	0.4095	0.4127	0.3421
	Total	**0.8420**	0.4956	0.4690	**0.5413**	0.4368	0.4721	0.4810
VAR	0–3	0.7561	0.4129	0.4907	0.4509	0.5667	**0.5527**	0.3712
	3–6	**0.8550**	0.4527	0.4507	0.3631	0.4707	0.1161	0.3671
	> 6	0.7906	0.4183	0.4589	0.2799	0.3129	0.2858	0.3254
	Total	0.8319	**0.6000**	0.5071	0.3657	0.5711	**0.4768**	0.3498
GAR	0–3	**0.8051**	**0.5101**	**0.6009**	0.4088	**0.5984**	0.4458	0.4488
	3–6	0.8193	0.4729	**0.5907**	**0.4479**	**0.5526**	0.4018	**0.5246**
	> 6	**0.8141**	**0.5096**	**0.6210**	**0.4329**	**0.5723**	**0.4313**	**0.4797**
	Total	0.8181	0.5139	**0.6050**	0.4629	**0.5753**	0.4316	**0.5160**

The number in bold indicates the maximum value in a certain horizon and age group. ‘Total’ means the total number of cases of EV-A71 subtype in Beijing.

For the performances of the three deep learning models in short-term or long-term predictions, the accuracies of CNNRNN-Res and RNN were relatively stable as the increase of horizon, except when the horizon was 4 months, while the accuracies of CNNRNN decreased when the horizons were 4, 6 and 8 months. In Table 1, the CNNRNN-Res model had the highest accuracies in almost all three age groups for forecasts of 1 and 2 months ahead, as well as the total number of EV-A71 cases (0.9062 and 0.8467, respectively). As for the long-term prediction, CNNRNN-Res model and RNN model achieved the highest accuracies in different age groups for the forecast of 6, 8 and 10 months ahead. When the horizon was 12 months, the CNNRNN model had the highest accuracies in age groups of 3–6 and > 6 (0.8310 and 0.7523, respectively), while CNNRNN-Res had the highest accuracy in age groups of 0–3 (0.9032). But the gaps between CNNRNN-Res and CNNRNN were small.

Among the three regressive models, GAR model has shown stability and superiority than AR or VAR models in both short-term and long-term prediction. In Table 2, GAR had the highest R-squares for the forecast of 1 and 2 months ahead in age groups of 0–3 (0.8051 and 0.5101) and > 6 (0.8141 and 0.5096). As for the long-term prediction, GAR had the highest R-squares for prediction of 4, 8 and 12 months ahead. When horizon was 6 or 10 months, the GAR model didn’t differ significantly from the optimal model in terms of R-squares for all age groups.

As for the comparison of accuracy and robustness between three deep learning models and three regressive models, GAR model had better performances than RNN model in the age groups of 0–3 (0.6009) and > 6 (0.6210), as well as in the total number of cases (0.6050) when the horizon was 4 months. But in the other prediction horizons, the deep learning model (numbers in bold in Table 1) all achieved higher R-squares than the regressive model (numbers in bold in Table 2) in the given age groups. Moreover, the performances of three deep learning models didn’t have a sharp decline in the long-term prediction, e.g. the R-squares of deep learning models only changed from 0.9062, 0.8845 and 0.8911 (horizon = 1 month) to 0.8538, 0.8661 and 0.7722 (horizon = 12), respectively, for the prediction of total number of cases. However, the performances of three regressive models declined gradually as the increase of horizon, e.g. the R-squares of them declined from 0.8420, 0.8319 and 0.8181 (horizon = 1 month) to 0.4810, 0.3498 and 0.5160 (horizon = 12), respectively, for the prediction the total number of cases. In order to visualize their differences on robustness, predictions of six models on the test set (horizon = 1 and 12 months) can be found as Supplementary Figure S1 online.

The differences and connections of outbreak trends among three age groups were also reflected. At every given horizon in Table 1, each deep learning model had similar R-squares in three age groups. In Table 2, GAR had similar accuracies in three age groups at each horizon, but performances of AR and VAR were not stable enough for different age groups. For example, when the horizon was 6 months, the AR model had the highest accuracies in the age group of 0–3 (0.5693) and the total number of cases (0.5413), but the AR model had the lowest accuracies in the same horizon for age groups of 3–6 (0.3501) and > 6 (0.2717). Such gaps of R-squares in three age groups still appeared in the VAR model when the horizon was 10 months.

### Accuracy assessment on peak intensity

**Figure 2:**
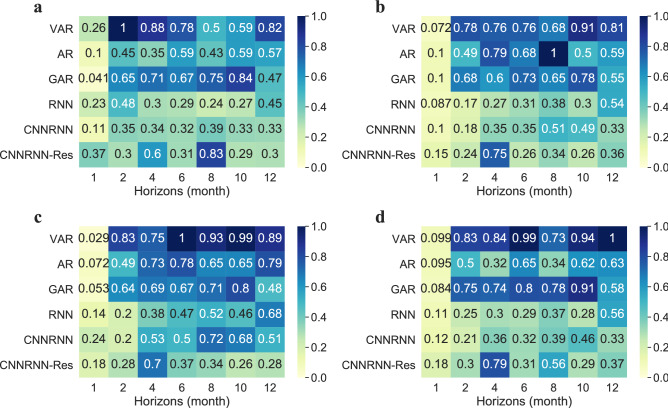
The normalized mean absolute errors (NMAE) of peak intensity forecasting. (**a**) Errors in age group of 0–3; (**b**) errors in age group of 3–6; (**c**) errors in age group of > 6; (**d**) errors of total number of cases.

The peak intensity of the EV-A71 subtype refers to the highest value of the curve each year. Peak intensity determines the level of early warning and the number of resources to prepare for the peak outbreak. Therefore, the prediction accuracy on peak intensity need to be analyzed separately from the overall point forecasts. Figure 2 shows the normalized mean absolute errors (NMAE) between the predicted peak intensities and the true peak intensities on the test set. They were normalized by maximum errors of 1,880.46, 2,791.61, 614.02 and 4,671.82, corresponding to the three age groups and the total number of cases, separately.

Three deep learning models had better accuracy and robustness on peak intensity forecast than three regressive models. The NMAE of three regressive models was higher than three deep learning models in Fig. 2a, b, c, d. The general NMAE of VAR was the largest, followed by the GAR. The NMAE of AR varied considerably with horizons, without a clear trend (Fig. 2b, d). As for the deep learning models, they performed better on peak intensity forecast, and with the increase of the horizon, the NMAE of deep learning models didn’t have an upward tendency. On the contrary, the NMAE of the three autoregressive models increased rapidly after horizon = 2.

NAME of peak intensity predictions varied in three age groups. VAR, RNN and CNNRNN models had increased NMAE in the age group of > 6. Deep learning models had no significant advantages in the age group of > 6. The models’ prediction ability of peak intensity is not completely consistent with their point prediction ability.

### Accuracy assessment on peak month

**Figure 3:**
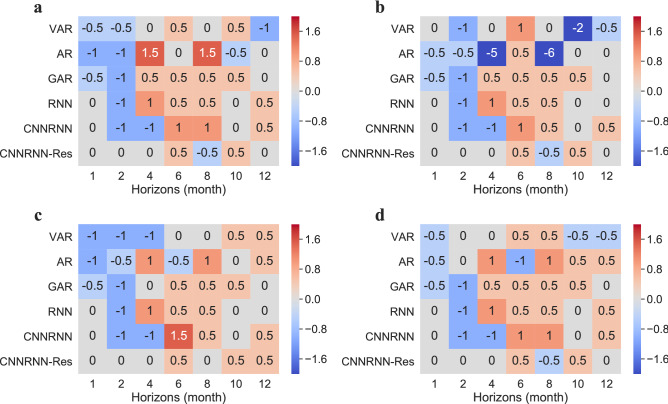
Average errors of peak month prediction. (**a**) Errors in the age group of 0–3; (**b**) errors in the age group of 3–6; (**c**) errors in the age group of > 6; (**d**) errors of the total number of cases.

Peak month is the time when the number of cases reaches the maximum in a year. A more accurate prediction on peak month will help the department of public health give early warning at the right time and reasonably arrange the prevention and control schedule. Figure 3 gives the average delay between the predicted peak month and the true peak month on the test set. Most of the average delays are between − 2 and 2, except for two outliers in Fig. 3b. A negative number represents a lagged prediction, while a positive number represents the prediction is ahead of the real peak month.

CNNRNN-Res model had the highest accuracies in peak month prediction in all age groups, while the AR model had the lowest accuracies, especially when the horizon was 4 or 8 months (Fig. 3a, b). Many models, except for the CNNRNN-Res model, gave lagged predictions (− 0.5 or − 1) when the horizon was 1 or 2 months. While in the long-term forecast, the predicted peak months were always ahead of the true values (Fig. 3a–d). The three age groups and the group of total number represented similar pattern on distribution of errors of peak month prediction.

### Forecast outcome of CNNRNN-Res model

**Figure 4:**
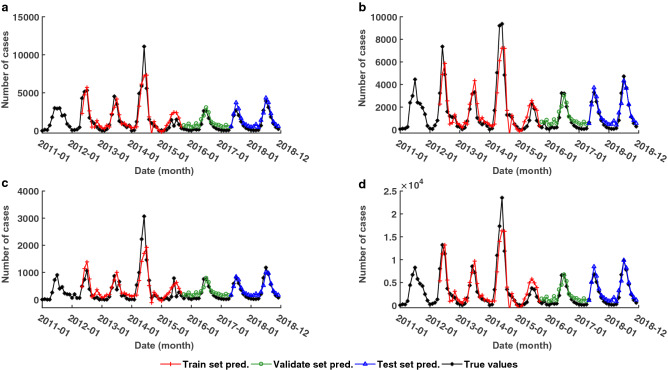
Prediction results of the CNNRNN-Res model in three age groups and the total number of cases (horizon = 1 month). (**a**) Prediction in the age group of 0–3; (**b**) prediction in the age group of 3–6; (**c**) prediction in the age group of > 6; (**d**) prediction of the total number of cases. Red cross dots represent prediction on train set; green dots represent prediction on validation set; blue triangles represent prediction on the test set and black dots represent true values.

The graphs in Fig. 4 show the predicted results of the CNNRNN-Res model with the horizon = 1 month. CNNRNN-Res model had a robust skill for point forecast and peak month forecast. Its prediction successfully captured the trend in all three age groups and the total number of cases, even though their peak shapes and magnitudes are varied. However, the CNNRNN-Res model couldn’t predict the troughs between two peaks, e.g. the trough in June 2015 (Fig. 4a) and the trough in July 2013 (Fig. 4c), because the model didn’t learn the existence of twin peaks from historical information. Similarly, there was an abnormal outbreak of EV-A71 subtype in 2014, and the predicted peak values in all age groups (Fig. 4a–c) and the predicted total number of cases (Fig. 4d) were lower than the true values. As for the long-term forecasting, predictions (horizon = 12 months) of GAR and CNNRNN-Res can be found as Supplementary Figure S2 online.

## Discussion and conclusions

HFMD is a childhood disease and has periodic outbreaks in Beijing every year. Regressive models were the classical methods for HFMD prediction, and the prediction of the incidences of different age groups had always been ignored. In this work, we compared the long-term and short-term prediction effects of the deep learning models (CNNRNN-Res, CNNRNN and RNN) and regression models (AR, VAR and GAR) for EV-A71 subtype in Beijing from 2011 to 2018. We divided the affected population into three age groups (0–3, 3–6 and > 6 years old) for simultaneous prediction, and analyzed the differences and connections among different age groups.

We evaluated the prediction performances using three metrics: R-squares of point forecast, NMAE of peak intensity forecast and average delays of peak month forecast. Deep learning models had better accuracy on point forecast in both short-term and long-term predictions, especially the CNNRNN-Res model. It’s worth noticing that the RNN model and CNNRNN-Res model were robust in long-term prediction. With the increase of prediction horizons, their R-squares only decreased slightly (Table 1), while the R-squares of regressive models decreased sharply (Table 2). The three deep learning models also maintained better accuracies in peak intensity prediction, but their advantages were not obvious in the age group of > 6 (Fig. 2c). In the prediction of peak month, all models had advanced or lagged errors (Fig. 3). There were mainly lagged errors in short-term prediction and advanced errors in long-term prediction. CNNRNN-Res model still had the highest skill for peak month prediction, indicating that it could respond to the changes in the curve faster. Overall, in our three tasks, the CNNRNN-Res model showed the best level of prediction.

The successes of the three deep learning models are attributed to their model capacities. AR, VAR and GAR models are essentially linear models, which make predictions by the weighted sum of history signals within the window length. The historical information will be forgotten after several iterations. On the contrary, a multi-layer deep learning model can fit complex nonlinear dependencies. More hidden layers or neurons will increase the representation space. Especially, each neuron of the RNN module has a mechanism of updating and resetting, and their cascade structure allows RNN to retain the long-term dependencies of history inputs.

It is necessary to divide the cases of EV-A71 subtype into different age groups for prediction because there are significant differences in the social contact networks and immunities of susceptible children. Children of 0–3 years old are children with a simple social network, while children of 3–6 years old are kindergarten children, and > 6 years old are elementary school students, with an expanded social network. Children over 6 years of age have higher immunity to EV-A71 than children under 6 years of age. It can be found from Fig. 1 that the outbreaks of three age groups are consistent in the time dimension and the scales of the outbreak in age groups of 0–3 and 3–6 are almost the same. The prediction errors of different age groups also share similar patterns (Figs. 2 and 3). These analyses indicate that the scale of the EV-A71 outbreak is more affected by children's immunity and seasonal factors.

Error indicators for infectious disease prediction should differ from those in other fields. Deep learning was implied earlier in fields such as finance, energy, and traffic prediction, and then introduced into the field of infectious disease prediction. The most used error indicator is the overall points prediction accuracy, but the indicators for infectious disease prediction should serve the practical applications, and narrow the gaps between modeling and model users. For example, the ‘FluSight’ challenge^25^ of the US evaluates the proposed models on future incidence prediction, peak intensity prediction, peak week prediction and onset week prediction, because these error indicators are directly related to the development of control measures by the public health department. Previous HFMD prediction^12–14,26–29^ didn’t use similar indicators. In our study, we evaluated our models on future point prediction, peak intensity prediction and peak month prediction, and these error indicators may facilitate deep learning models to be more widely used in the practice of epidemic prediction. Our study still has some limitations. First, we can’t explain more specifically why deep learning models are dominant, because interpretability of deep learning is a long-standing problem that has not been solved yet. Second, our data set is limited in length and the conclusions reached may not be general. To measure how the characteristics of deep learning models scale with data accuracy and quantity, a lot of experiments and simulations need to be done^30^.

The main conclusions of this paper are threefold. Firstly, deep learning models have higher precision than regressive models in EV-A71 outbreak predictions. Secondly, three deep learning models, especially the CNNRNN-Res model, are more robust in long-term predictions. Thirdly, the number of cases of three age groups are consistent in time, but there is heterogeneity in the peak intensity in age groups of > 6. We believe that deep learning models and practical error indicators should be more widely used in the prediction and early warning of infectious diseases, and the consistency of the incidence patterns of multiple age groups could be utilized to improve the prediction accuracy.

The continuity of research in the future includes the following. First, investigate the performance of deep learning models combining with seasonal factors and web search indexes in HFMD prediction. Because seasonal changes are the main factors affecting the incidences, and the search indexes are helpful for real-time forecasting. Second, ensemble predictions need more attention. Results in Tables 1 and 2 indicate that different models have varied forecasting advantages. The fusion of deep learning model and regressive model may improve the prediction accuracy and robustness.

## Methods

### Structures of CNNRNN-Res, CNNRNN and RNN models

**Figure 5:**
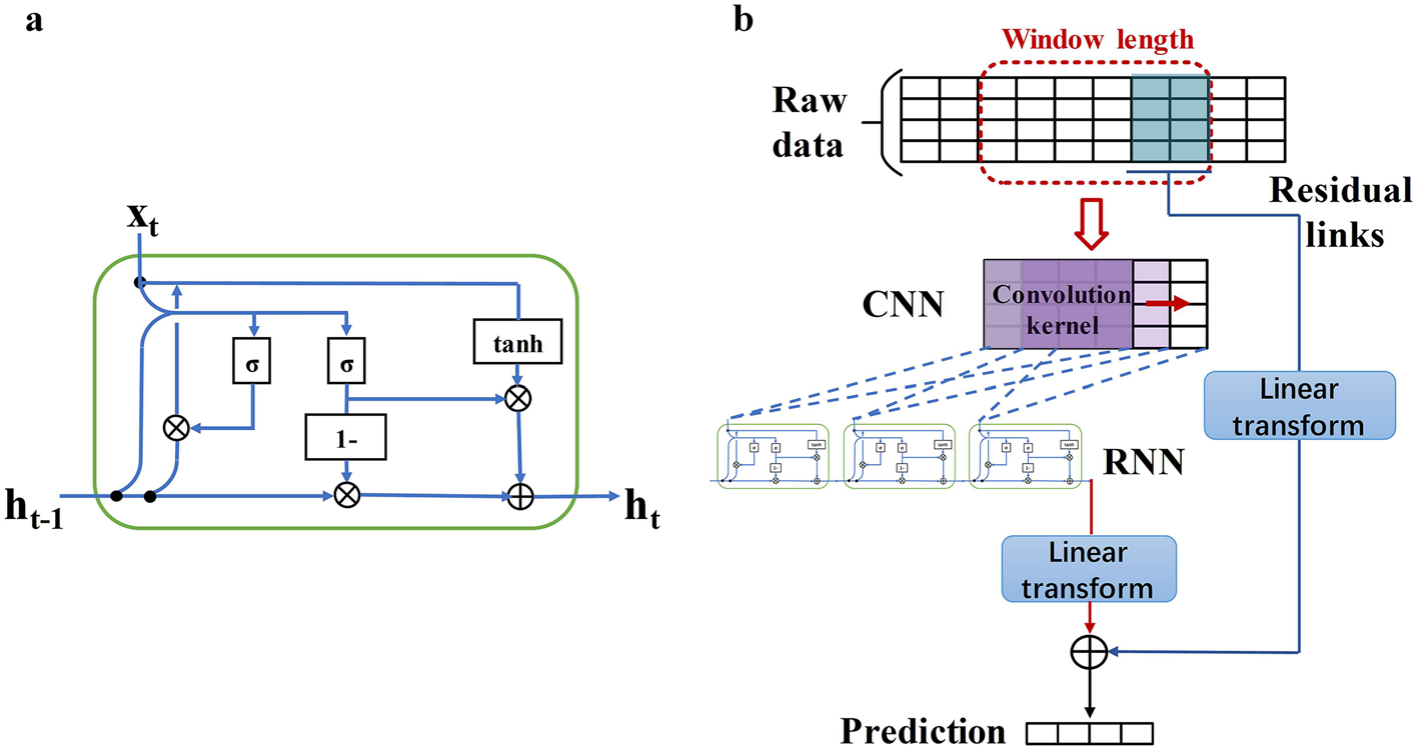
Structural diagrams of (**a**) gated recurrent unit (GRU) cell and (**b**) the CNNRNN-Res model.

Researchers at Carnegie Mellon University proposed and shared the source codes of CNNRNN-Res model^18^. CNNRNN-Res model is mainly composed of three parts (Fig. 5b): CNN module, RNN module and residual links. The other two ablation models, CNNRNN and RNN, are obtained after removing some functional modules of CNNRNN-Res, i.e., the CNNRNN model doesn’t have residual links, and RNN only uses the RNN module to make a prediction.

CNN module captures the correlation among the three age groups using convolution operation. The convolution kernel is a two-dimensional weight matrix. This matrix and the input time-sequence segments within the window length are convolved to obtain the time-series features. The output of the CNN module is used as the input of the RNN module. There are many variations of the RNN module, and the gated recurrent unit (GRU) is used in this research. Figure 5a is a single GRU cell and it computes the following function:

1 $$ r_{t} = \sigma \left( {W_{ir} x_{t} + b_{ir} + W_{hr} h_{{\left( {t - 1} \right)}} + b_{hr} } \right) $$

2 $$ z_{t} = \sigma \left( {W_{iz} x_{t} + b_{iz} + W_{hz} h_{{\left( {t - 1} \right)}} + b_{hz} } \right) $$

3 $$ n_{t} = \tanh \left( {W_{in} x_{t} + b_{in} + r_{t} {*}\left( {W_{hn} h_{{\left( {t - 1} \right)}} + b_{hn} } \right)} \right) $$

4 $$ h_{t} = \left( {1 - z_{t} } \right){*}n_{t} + z_{t} {*}h_{{\left( {t - 1} \right)}} $$

where $h_{t}$ is the hidden state at time $t$, $x_{t}$ is the input data at time $t$, $h_{{\left( {t - 1} \right)}}$ is the hidden state at time $t - 1$ or the initial hidden state at time $0$, and $r_{t}$, $z_{t}$, $n_{t}$ are the reset, update, and new gates, respectively. $\sigma$ is the sigmoid function, and ${*}$ is the Hadamard product. The internal parameters of the three deep learning models are fine-tuned by means of back-propagation of prediction errors.

### Constructing the AR, VAR and GAR models

AR, VAR and GAR are classical regression methods in time series prediction. AR treats the age groups independently, i.e. assume the numbers of cases in different age groups are not correlated. AR can be formalized as:

5 $$ x_{t + h}^{\left( i \right)} = \mathop \sum \limits_{p = 0}^{w - 1} \alpha_{p}^{\left( i \right)} x_{t - p}^{\left( i \right)} + \varepsilon_{t + h} + c^{\left( i \right)} $$

where *p* is the order of AR, $w$ is the input window length, $h$ is the prediction horizon, $x_{t}^{\left( i \right)}$ is the *i-*th input variable at time *t*, and $\alpha_{p}^{\left( i \right)}$ is the weight parameter. $\varepsilon_{t + h}$ is random noise at time *t* + *h*, and $c^{\left( i \right)}$ is the intercept term. GAR simplifies the AR model. Its premise assumes that the variation pattern of each age group is consistent, and the same set of $\alpha_{p}$ and ${\text{c}}$ can be used to predict the number of cases in different age groups.

VAR fits the cross-signal dependencies and it is more complex and expressive. The VAR model assumes that the number of patients in each age group is correlated with those in the rest groups, which is different from the independent hypothesis of AR and the consistent variation hypothesis of GAR. VAR can be formalized as:

6 $$ \tilde{\user2{x}}_{t + h} = \mathop \sum \limits_{p = 0}^{w - 1} A_{p} {\varvec{x}}_{t - p} + {\varvec{\varepsilon}}_{t + h} + {\varvec{c}} $$

where $A_{p}$ is the parameter matrix to capture correlations among age groups.

### Hyper-parameters selection and model training

We implemented the six models using Facebook's open-source platform–Pytorch. The original dataset was divided into a training set, validation set and test set, as described in the results section. Before training, we need to set up some hyper-parameters, as they determine the structure of models and can’t be changed through training. We set up an optional set for each hyper-parameter, i.e. the number of hidden neurons (5, 10, 20 or 40), the length of the input window (2, 4, 8, 16 or 32), residual window (4, 8 or 16), prediction horizons (1, 2, 4, 6, 8, 10, 12 months) and the residual ratio (0.01, 0.1, 0.5 or 1). Then, we conducted a grid search over all hyper-parameters and fine-tuned these six models using the training set, separately. After that, we put validation set into the trained models, and got six optimal models that had the highest R-squares of points forecasts on the validation set. The optimal combination of hyper-parameters for each model can be found as Supplementary Table S1 online.

### Metrics of prediction errors

The six optimal models forecasted on test set with seven prediction horizons (horizon = 1, 2, 4, 6, 8, 10, 12 months). Three metrics were implied to measure prediction accuracies of three deep learning models and three regressive models: R-squares of point forecasts, NMAE on peak intensity forecasts, and average delays of peak month forecasts. R-squares performed an overall prediction accuracy evaluation on all discrete points in the test set. Because the predicted peak intensity and peak time have more practical guiding significance, NMAE only measures the peak intensity of each year, and the average delays only focus on when the peak month arrives. The formulas of three metrics are as follows:

7 $$ R_{j,h}^{2} = 1 - \frac{{\mathop \sum \nolimits_{i = 1}^{20} \left( {\hat{y}_{i}^{j,h} - y_{i} } \right)^{2} }}{{\mathop \sum \nolimits_{i = 1}^{20} \left( {\overline{y}_{i} - y_{i} } \right)^{2} }} $$

8 $$ NMAE^{j,h} = \frac{{\left| {y_{peak} - \hat{y}_{peak}^{j,h} } \right|}}{{\mathop {\max }\limits_{h} \left( {\left| {y_{peak} - \hat{y}_{peak}^{j,h} } \right|} \right)}} $$

9 $$ delay^{j,h} = t_{peak} - \hat{t}_{peak}^{j,h} $$

where $$j $$ represents the age groups, $$h$$ represents different prediction horizons, $$y$$ is the number of cases in original dataset, and $$\hat{y}$$ is the predicted value. The three metrics corresponding to different horizons are used to compare the performance of the short-term and long-term predictions of the models. The three metrics corresponding to different age groups are used to analyze the outbreak patterns of HFMD in different age groups.

### Ethics statement

All methods were carried out in accordance with the principles of the Declaration of Helsinki. The experimental protocols were approved by the ethical committee of Beijing Center for Diseases Prevention and Control. And informed consents were obtained from all participants and their legal guardians. All records were anonymized and no individual information can be identified.

## Supplementary information

Supplementary information

## Data Availability

Please contact corresponding author for data requests.
