# Supplementary material for: Using deep learning to predict the hand-foot-and-mouth disease of enterovirus A71 subtype in Beijing from 2011 to 2018
Source: Sci Rep. 2020 Jul 22;10:12201. doi: 10.1038/s41598-020-68840-3 (PMC7376109; doi:10.1038/s41598-020-68840-3)
Supplement: Supplementary file 1 — Supplementary information [file 41598_2020_68840_MOESM1_ESM.pdf]

Using Deep Learning to Predict the Hand-Foot-and-Mouth Disease of Enterovirus A71 Subtype in Beijing from 2011 to 2018

Authors:

Yuejiao Wang, graduate student, Institute of Automation Chinese Academy of Sciences, Beijing, China; University of Chinese Academy of Sciences, Beijing

Zhidong Cao, PhD, Institute of Automation, Chinese Academy of Sciences, Beijing, China

Daniel Zeng, PhD, IEEE fellow, AAAS fellow, Institute of Automation, Chinese Academy of Sciences, Beijing, China

Xiaoli Wang, PhD, Institute for Infectious Disease and Endemic Disease Control, Beijing Center for Disease Prevention and Control, Beijing, China

Quanyi Wang, PhD,Institute for Infectious Disease and Endemic Disease Control, Beijing Center for Disease Prevention and Control, Beijing, China

Corresponding author: Zhidong Cao, No. 95, Zhongguancun East Road, Haidian District, Beijing, China; email: zhidong.cao@ia.ac.cn; phone number: +86-10-82544716.

Supplementary Figure and Table Legends

Table S1. Optimal hyper-parameters of AR, VAR, GAR, RNN, CNNRNN and CNNRNN-Res in different prediction horizons.

Figure S1. Predictions of six models of EV-A71 subtype in Beijing on test set. (a) Prediction of 0-3 years old (horizon=1 month). (b) Prediction of 0-3 years old (horizon=12 months). (c) Prediction of 3-6 years old (horizon=1 month). (d) Prediction of 3-6 years old (horizon=12 months). (e) Prediction of above 6 years old (horizon=1 month). (f) Prediction of above 6 years old (horizon=12 months). (g) Prediction of total number (horizon=1 month). (h) Prediction of total number (horizon=12 months).

Figure S2. Predictions of GAR model and CNNRNN-Res model on EV-A71 subtype in Beijing from 2011 to 2019 (horizon = 12 months). (a) Prediction of 0-3 years old from GAR. (b) Prediction of 0-3 years old from CNNRNN-Res. (c) Prediction of 3-6 years old from GAR. (d) Prediction of 3-6 years old from CNNRNN-Res. (e) Prediction of above 6 years old from GAR. (f) Prediction of above 6 years old from CNNRNN-Res. (g) Prediction of total number from GAR. (h) Prediction of total number from CNNRNN-Res.

Table S1.

Prediction horizon (month)	Models	Residual ratio	Length of residual window	Length of input window	Hidden neurons
1	AR	--	--	8	--
	VAR	--	--	4	--
	GAR	--	--	4	--
	RNN	--	--	8	5
	CNNRNN	--	--	8	5
	CNNRNN-Res	0.01	8	16	10
2	AR	--	--	16	--
	VAR	--	--	16	--
	GAR	--	--	16	--
	RNN	--	--	4	40
	CNNRNN	--	--	4	40
	CNNRNN-Res	0.01	4	16	20
4	AR	--	--	8	--
	VAR	--	--	16	--
	GAR	--	--	32	--
	RNN	--	--	4	40
	CNNRNN	--	--	2	20
	CNNRNN-Res	0.01	16	16	5
6	AR	--	--	8	--
	VAR	--	--	8	--
	GAR	--	--	8	--
	RNN	--	--	8	5
	CNNRNN	--	--	8	5
	CNNRNN-Res	0.01	4	16	5
8	AR	--	--	8	--
	VAR	--	--	8	--
	GAR	--	--	8	--
	RNN	--	--	8	5
	CNNRNN	--	--	8	5
	CNNRNN-Res	0.01	4	4	5
10	AR	--	--	4	--
	VAR	--	--	4	--
	GAR	--	--	4	--
	RNN	--	--	8	5
	CNNRNN	--	--	8	5
	CNNRNN-Res	0.01	4	4	5

Prediction horizon (month)	Models	Residual ratio	Length of residual window	Length of input window	Hidden neurons
12	AR	--	--	2	--
	VAR	--	--	2	--
	GAR	--	--	2	--
	RNN	--	--	4	5
	CNNRNN	--	--	2	5
	CNNRNN-Res	0.01	4	4	5

-- represents that the corresponding model doesn’t need this hyper-parameter

Figure S1.

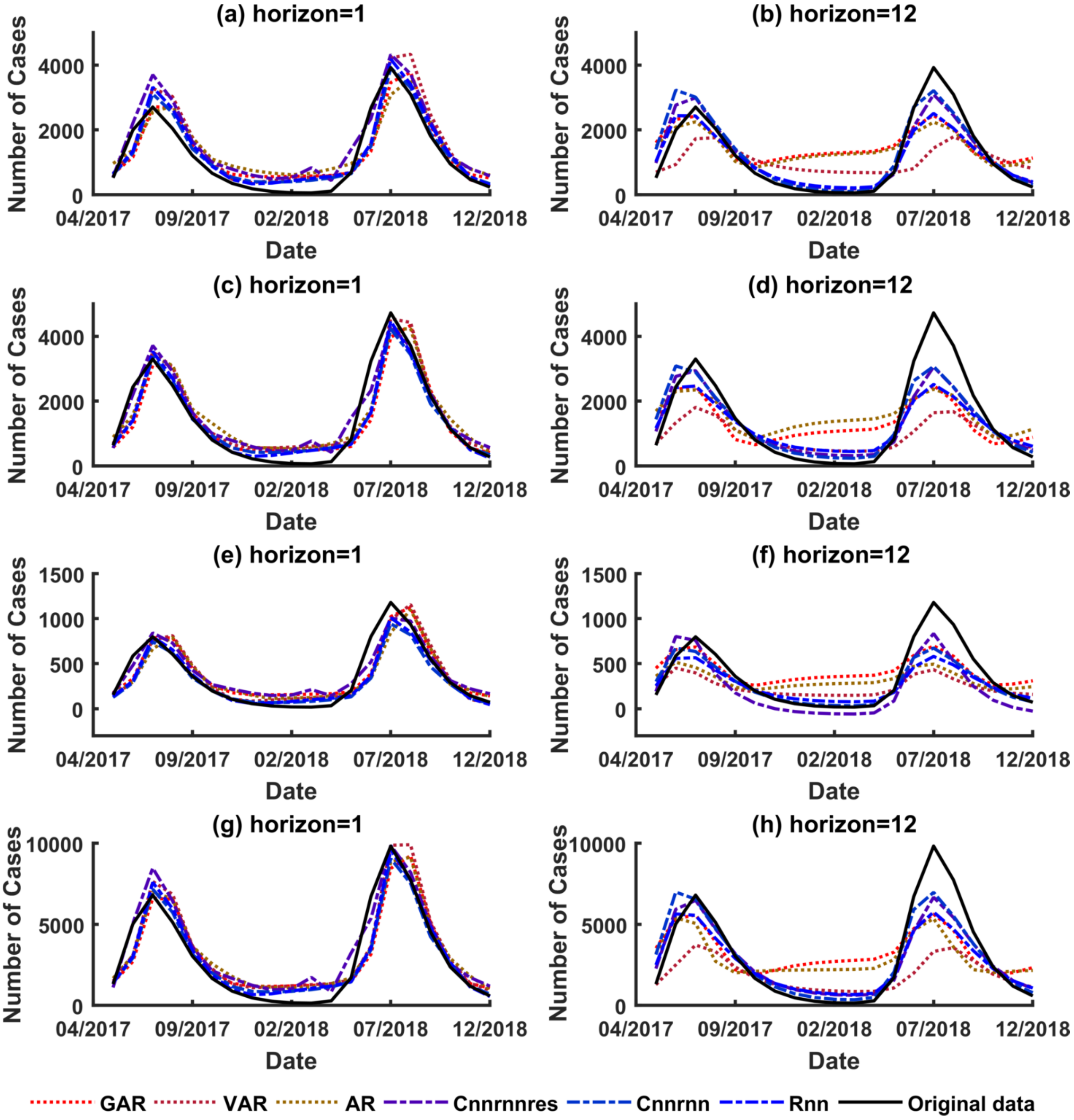
(a) horizon=1
(b) horizon=12
(c) horizon=1
(d) horizon=12
(e) horizon=1
(f) horizon=12
(g) horizon=1
(h) horizon=12
Number of Cases
Date
04/2017
09/2017
02/2018
07/2018
12/2018
0
500
1000
1500
2000
4000
5000
10000
GAR
VAR
AR
Cnnrnnres
Cnnrnn
Rnn
Original data

Figure S2.

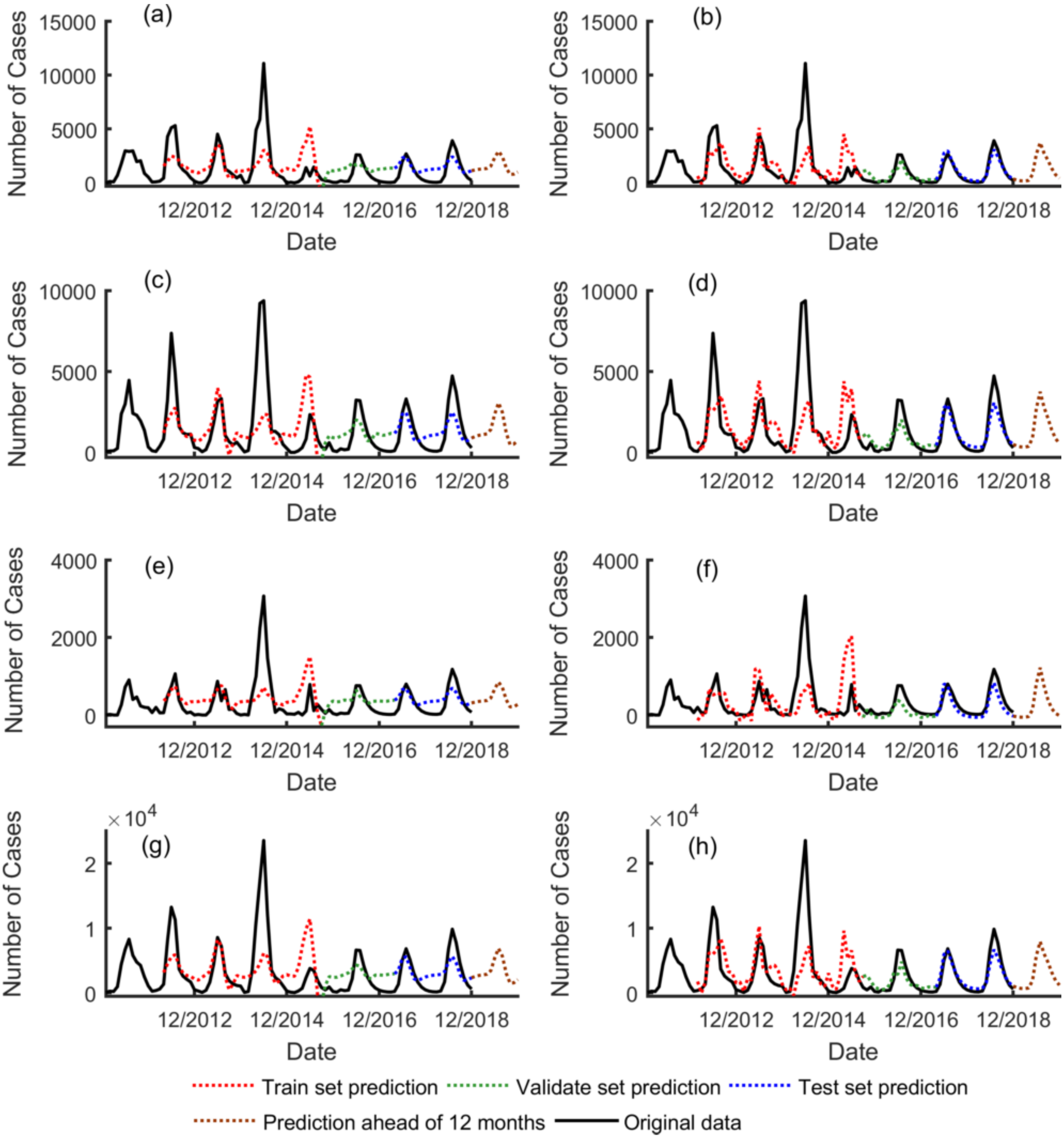

(a)
(b)
(c)
(d)
(e)
(f)
(g)
(h)
Number of Cases
Date
15000
10000
5000
4000
2000
$\times 10^4$
0
12/2012
12/2014
12/2016
12/2018
Train set prediction
Validate set prediction
Test set prediction
Prediction ahead of 12 months
Original data
